# Conditioning by a Previous Experience Impairs the Rewarding Value of a Comfort Meal

**DOI:** 10.3390/nu15102247

**Published:** 2023-05-09

**Authors:** Adoracion Nieto, Dan M. Livovsky, Fernando Azpiroz

**Affiliations:** 1 Digestive System Research Unit, University Hospital Vall d’Hebron, 08035 Barcelona, Spain; 2 Departament de Medicina, Universitat Autònoma de Barcelona, Bellaterra, 08193 Cerdanyola del Vallès, Spain; 3 Centro de Investigación Biomédica en Red de Enfermedades Hepáticas y Digestivas (Ciberehd), Instituto de Salud Carlos III, 28029 Madrid, Spain; 4 Digestive Diseases Institute, Shaare Zedek Medical Center, Faculty of Medicine, Hebrew University of Jerusalem, Jerusalem 9103102, Israel

**Keywords:** Pavlovian conditioning, aversive conditioning, eating behaviour, digestive sensations, postprandial symptoms, digestive well-being, food valence

## Abstract

Background. Meal ingestion induces a postprandial experience that involves homeostatic and hedonic sensations. Our aim was to determine the effect of aversive conditioning on the postprandial reward of a comfort meal. Methods: A sham-controlled, randomised, parallel, single-blind study was performed on 12 healthy women (6 per group). A comfort meal was tested before and after coupling the meal with an aversive sensation (conditioning intervention), induced by infusion of lipids via a thin naso-duodenal catheter; in the pre- and post-conditioning tests and in the control group, a sham infusion was performed. Participants were instructed that two recipes of a tasty humus would be tested; however, the same meal was administered with a colour additive in the conditioning and post-conditioning tests. Digestive well-being (primary outcome) was measured every 10 min before and 60 min after ingestion using graded scales. Results: In the aversive conditioning group, the comfort meal in the pre-conditioning test induced a pleasant postprandial experience, which was significantly lower in the post-conditioning test; the effect of aversive conditioning (change from pre- to post-conditioning) was significant as compared to sham conditioning in the control group, which showed no differences between study days. Conclusion: The hedonic postprandial response to a comfort meal in healthy women is impaired by aversive conditioning. ClinicalTrials.gov ID: NCT04938934.

## 1. Introduction

The digestive process that follows meal ingestion is associated with a postprandial experience that involves homeostatic sensations (satiety, fullness) with a hedonic dimension (digestive well-being, mood) [1]. The postprandial experience depends on the characteristics of the meal (organoleptic, amount and composition) and of the individual, including digestive function, intestinal sensitivity and cognitive/emotive factors, which may be influenced by a variety of conditions [2]. Pavlovian conditioning, also known as classical conditioning, refers to the behavioural technique of pairing a physiological stimulus with a neutral stimulus; repeated exposure to the pairing induces a learning process, by which the biologic response to the physiological stimulus is triggered by the neutral stimulus alone. Pavlovian conditioning has been shown to magnify the expectation of aversive sensations and has been postulated as a mechanism of hypervigilance and visceral hypersensitivity [3,4,5,6]. Associative learning, understood as the learned association between two unrelated stimuli, has also been also shown to induce taste aversion and avoidance: e.g., a pleasant taste becomes disagreeable by previous association with an unpleasant experience [7]. 

We hypothesised that the postprandial experience, in particular the hedonic component (i.e., postprandial sensation of digestive well-being), may be modified by conditioning. Our specific aim was to determine the effect of aversive conditioning on the hedonic and homeostatic sensations in response to a comfort meal in healthy subjects. 

The postprandial experience in humans is important because it may influence dietary decisions and habits. Moreover, a negative postprandial experience is a main complaint in patients with functional gut disorders, particularly in those with functional dyspepsia [8,9]; hence, aversive conditioning might be a mechanism of meal intolerance and postprandial symptoms in these patients.

## 2. Material and Methods

### 2.1. Experimental Design

A sham-controlled, randomised, parallel, single-blind study on the effect of aversive conditioning on the responses to a comfort meal in healthy women was performed in a tertiary referral centre between February and August 2021. The research was conducted according to the Declaration of Helsinki. The protocol for the study had been previously approved by the Institutional Review Board of the University Hospital Vall d’Hebron (Comitè d’Ètica d’Investigació Clinica, Vall d’Hebron Institut de Recerca; protocol number PR(AG)338/2016M approved 28 October 2016, revised 11 December 2020) and all participants provided written informed consent. The study protocol was registered with ClinicalTrials.gov NCT04938934. 

### 2.2. Participants

Twelve, non-obese, non-dieting and weight-stable women (6 per group), without history of gastrointestinal symptoms were recruited by public advertising to participate in the study. For this pilot, proof-of-concept study, only women were included for the sake of homogeneity and because some data indicate that they are more susceptible to factors that modulate the postprandial experience than men [10]. Exclusion criteria were chronic health conditions, previous abdominal surgery (except appendectomy or hernia repair), use of medications (except occasional use of NSAIDs and antihistamines), alcohol abuse and use of recreational drugs. By specific questioning, candidates with a history of anosmia or ageusia, antecedents of obesity (defined as body mass index > 30 kg/m^2^), current dieting or any pattern of selective eating, such as vegetarianism, were not included in the study to prevent potential biases on the responses to food ingestion. Candidates were asked whether they liked hummus, and those who did not were not included. Absence of current digestive symptoms was verified using a standard abdominal symptom questionnaire (no symptom > 2 on a 0–10 scale). Psychological and eating symptoms and/or traits were evaluated using the Hospital Anxiety and Depression Scale (HAD), Dutch Eating Behaviour Questionnaire (DEBQ—Emotional eating, External eating, Restrained eating) and Physical Anhedonia Scale (PAS); participants were not included if they scored >7 on the anxiety or depression subscales [11]; cut-offs for emotional eating (>2.83), external eating (>3.5) and restrained eating (>3.0) were adapted from a study in the local population [12]. Studies were performed during the follicular phase of the menstrual cycle (days 5–15). For this pilot proof-of-concept study, no a priori sample size calculation was performed; analysis of the data performed after 12 studies were completed indicated that a sample size of 8 subjects (i.e., 4 per group) was required to detect changes in the primary outcome with 90% power and a 5% significance threshold, and hence, no further participants were included. Thus, each group consisted of 6 participants.

### 2.3. Experimental Paradigm

**Figure 1:**
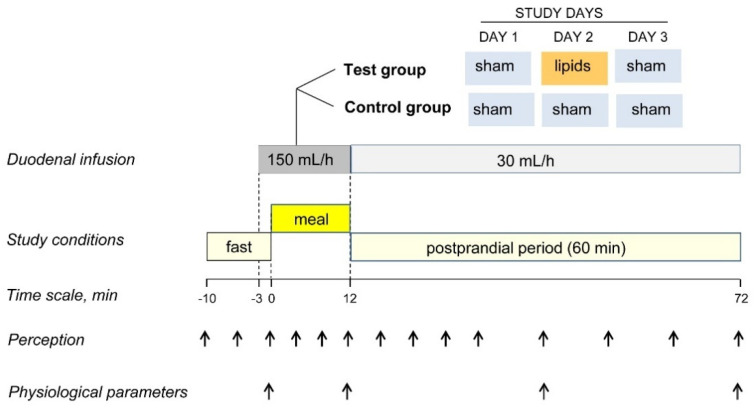
Experimental design and procedure. In a sham-controlled, parallel, randomised, blind study, a comfort meal was paired with duodenal lipid infusion to induce aversive conditioning (DAY 2) and the responses to the meal were compared before (DAY 1) and after conditioning (DAY 3).

Participants were informed that the aim of the study was to investigate the effect of meal composition on the postprandial responses and that a nasoduodenal tube was used to evaluate gastric outflow. Participants were informed that two recipes of a tasty humus with different compositions would be tested; however, the same meal (low-fat humus) without or with the addition of a colourant (i.e., non-coloured or coloured) was administered (Figure 1). Using a computerised random sequence generator, participants were allocated into aversive conditioning (intervention) or sham conditioning (control) groups. During meal ingestion, either lipids or sham infusion was simultaneously infused single-blind (without participants knowing which) into the duodenum via the nasoduodenal catheter (see below). Each participant underwent three experiments on consecutive days, as follows (Figure 1). First day—pre-conditioning exposure: non-coloured meal plus sham infusion in both groups. Second day—conditioning intervention: coloured meal in both groups plus (a) duodenal lipid infusion in the aversive conditioning group (to induce aversive sensations, e.g., a negative sensation of digestive well-being) or (b) sham infusion in the sham conditioning (control) group. Third day—post-conditioning exposure: coloured meal plus sham infusion in both groups. Primary outcome: effect of conditioning on digestive well-being measured by scales (difference in the area under the curve from pre-conditioning to post-conditioning, i.e., day 3 minus day 1) in aversive conditioning versus sham conditioning groups.

### 2.4. General Procedure

During the 3 consecutive study days, participants were instructed to refrain from strenuous physical activity, to consume a standard dinner (100 g chicken, 50 g rice, 50 g white bread and one apple; 503 kcal, 7 g fat, 82 g carbohydrates, 30 g protein) the night before, to fast overnight and to eat a standard breakfast (200 mL coffee with semi-skimmed milk and a 50 g white bread sandwich with 30 g ham and 40 g cheese; 338 kcal, 11 g fat, 38 g carbohydrate, 24 g protein) 4 h before each study. After intubation per nose, the catheter (Flocare Bengmark NI Tube, Nutricia Medical, Hoofddorp, The Netherlands) was positioned into the duodenum under fluoroscopic control. Studies were conducted in a quiet, isolated room. Outcomes were measured 10 min before ingestion of the probe meal (pre-ingestion period), during the ingestion period and during the 60 min after ingestion (postprandial period) (Figure 1).

### 2.5. Interventions

#### 2.5.1. Probe Meal

The probe meal consisted of 150 g low-fat hummus (219 Kcal; 12 g fat, 82 g carbohydrates, 13 g protein; Hummus Classic, Ametller Origen, Barcelona, Spain) served at a controlled temperature (20 °C), 20 g toasts (81 Kcal; 0.9 g fat, 15.2 g carbohydrates, 2.4 g protein; Mini Tostas, Bimbo, Barcelona, Spain) and 120 mL water. The probe meal was administered stepwise in 3 equal servings at a fixed rate: every 180 s one meal portion (50 g hummus plus a 6.6 g toast) was presented on a tray; after each serving, participants were allowed 60 s for evaluation of digestive sensations (see below); total ingestion time was 12 min, the water load (120 mL) was ingested ad libitum throughout the ingestion period. On the 1st study day, the original humus preparation (i.e., non-coloured) was served; on the 2nd and 3rd study days (conditioning and post-conditioning experiments, respectively), the humus was coloured by adding 1% fat-soluble, odourless and flavourless pink colourant (Decora, Karma, Salerno, Italy), to modify its appearance, but not its organoleptic characteristics or nutrient composition. The composition and meal load were established based on a series of preliminary feasibility studies. 

#### 2.5.2. Duodenal Infusion

Aversive conditioning (2nd study day in aversive conditioning group only) was produced by infusion of lipids (300 mg/mL purified soybean oil; Intralipid, Fresenius Kabi, Barcelona, Spain) into the duodenum via the nasoduodenal catheter. Lipids were continuously infused starting 3 min before, during and 60 min after ingestion of the probe meal (total infusion time = 75 min) using an infusion pump (Compat Ella Push, Nestle Health Science, Barcelona, Spain) at a rate of 150 mL/h during the first 15 min (3 min pre-ingestion period and 12 min ingestion period) and at 30 mL/h during the 60 min postprandial period (Figure 1). On the rest of the study days (i.e., 1st and 3rd days in the conditioning group, and the three study days in the control group), a sham infusion was performed following the same procedure, but diverting the lipid flow from the infusion line via a 3-way stopcock to a reservoir. Lipid and sham infusions were performed single-blind, i.e., without the participants knowing the type of infusion.

The aversive conditioning procedure (lipid load and delivery rate) was established by a series of preliminary studies in 3 additional participants so that the lipid infusion would induce a negative sensation of digestive well-being (below score −2 on a −5 to +5 scale, see below) without severe nausea, bloating or pain (score ≤ 2 on 0–10 scales; see below). 

### 2.6. Outcomes

#### 2.6.1. Perception of Homeostatic and Hedonic Sensations

Five 10-cm scales graded from −5 to +5 were used to measure: (a) meal wanting (impossible/eagerly), (b) meal liking (very disagreeable/very agreeable), (c) hunger/satiety (extremely hungry/completely satiated), (d) digestive well-being (extremely unpleasant sensation/extremely pleasant sensation) and (e) mood (negative/positive); three additional 10-cm scales graded from 0 (not at all) to 10 (very much) were used to measure (f) abdominal bloating–fullness, (g) discomfort–pain and (h) nausea. The wanting scale was scored at the presentation of each meal serving (how much would you like to eat this portion) and at the end of the meal (how much would you like to eat another portion). The liking scale was scored after each meal serving (how much did you like eating the previous portion). The rest of the scales were scored: (a) during the pre-ingestion period (10 min before the meal) at 5 min intervals, (b) during meal ingestion, after each meal serving, and (c) during the postprandial period at 5 min intervals during the first 20 min and at 10 min intervals up to 60 min after ingestion (Figure 1). It has been previously shown that these scales detect consistent and reproducible differences in post-prandial sensations induced by various conditioning factors [13,14,15,16,17,18,19] and that perception measurements correlate with changes in circulating metabolites [20,21] and with some objective parameters of brain function measured by functional magnetic resonance [22,23].

#### 2.6.2. Physiological Parameters

The following physiological parameters were measured at 4 time points: before meal ingestion (baseline) and at the beginning, mid and end of the postprandial observation period (0 min, 30 min and 60 min after ingestion) (Figure 1).

Gastric emptying was measured by ultrasonography, as previously described [24,25]. In brief, ultrasound images of the gastric antrum were obtained using a Chison ultrasound scanner (ECO1; Chison, Wuxi, China) with an abdominal 3.5 Hz probe (C3A; Chison, Wuxi, China); images were obtained with the subjects seated and leaning slightly backwards in an ergonomic chair. Gastric images between antral contractions were obtained in triplicate; using the superior mesenteric vein and the aorta as landmarks, the outer profile and the cross-sectional area of the antrum were measured using the built-in calliper and measurement tool.

Changes in abdominal girth from pre-ingesta were measured by a tape measure placed over the umbilicus and the superior edge of the iliac crests [26]. The position of the tape was marked over the skin for subsequent measurements.

Changes in the position of the diaphragm from the pre-ingestion level were determined at each time point, as previously described [24]. Briefly, the position of the lower margin of the right liver lobe at the right anterior axillary line was identified by ultrasonography (Eco 1, Chison Medical Technologies, Wuxi, China) using a 3.5 MHz curved array transducer held over the edge of the costal wall in the coronal plane with the shaft held in a horizontal position and the head in an axial direction. At each time point, the position (averaged over 3 respiratory cycles) was marked over the skin.

### 2.7. Statistical Analysis

Calculations were performed using SPSS Statistics for Windows (Version 25.0, IBM Corp, Armonk, NY, USA). A significance level of 5% (two tails) was used for comparisons. 

In each group, the means and standard errors of the measured variables were calculated. In each experiment, the effects of the intervention on sensation scores were analysed, measuring the area under the curve normalised for baseline (except for the wanting and liking scores, which were not normalised) as follows: for each observation interval, the area was calculated as duration (min) of the observation interval × normalised score (absolute score—mean premeal score); the area under the curve during ingestion and the postprandial period (expressed as score × min) was calculated as the sum of the area of all observation intervals. 

In each participant, the effect of the aversive (or sham) stimulus was measured as the difference in the area under the curve on day 2 (duodenal lipids or sham infusion) minus day 1 (pre-conditioning); the effect of conditioning (previous exposure to aversive stimulus) was measured as the difference in day 3 (post-conditioning) minus day 1 (pre-conditioning). Mean values for the test group (lipid infusion) and control group (sham infusion) were calculated, and statistical analyses within groups and between groups were performed. The Shapiro–Wilk test was used to determine the normality of data distribution. Parametric normally distributed data were compared by Student’s *t*-test for paired or unpaired data; otherwise, the Wilcoxon signed-rank test was used for paired data, and the Mann–Whitney U test was used for unpaired data. Differences were considered significant at a *p* value < 0.05.

All co-authors had access to the study data and reviewed and approved the final manuscript.

## 3. Results

### 3.1. Demographics

Participants were 30.9 ± 2.3 years of age (range 23–49 years), had a 21.3 ± 0.5 kg/m^2^ body mass index (range 18.6–24.8 kg/m^2^), scored 11.8 ± 2.1 in the physical anhedonia scale (range 2–23) and were non-smokers. No differences between the aversive conditioning and control groups were detected. Intubation was well tolerated without side effects; all participants completed the studies and were included for analysis. 

### 3.2. Responses to the Probe Meal before Conditioning (Study Day 1)

**Figure 2:**
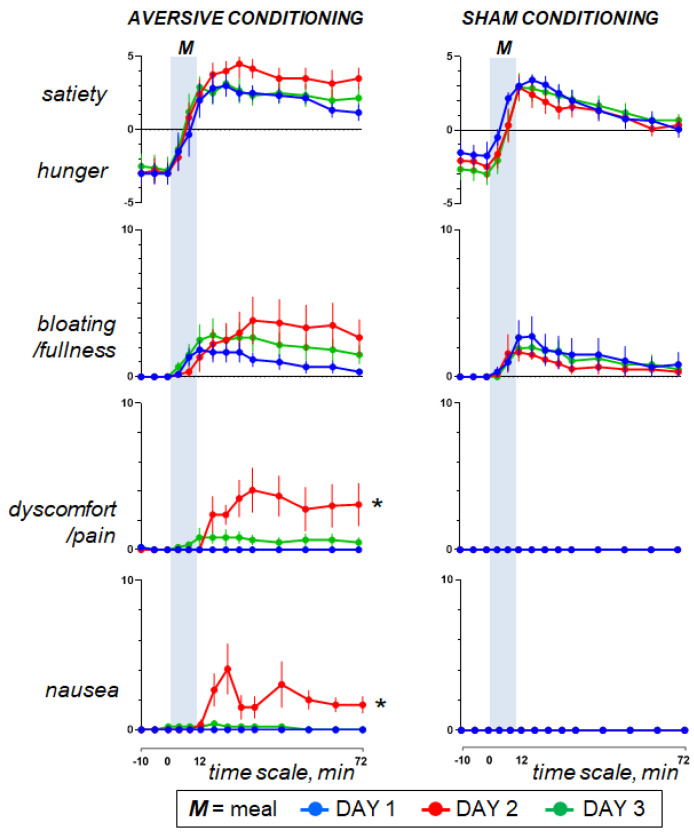
Homeostatic sensations. Concomitant duodenal lipid infusion on day 2 impaired the postprandial response and the effect was significant for abdominal discomfort and nausea (effect measured as the change in the area under the curve on day 2 minus day 1; * *p* = 0.002 vs. sham infusion). However, data on day 3 show that aversive conditioning in the test group (duodenal lipid infusion on the previous day) did not induce significant effects. Values represent mean ± SE.

**Figure 3:**
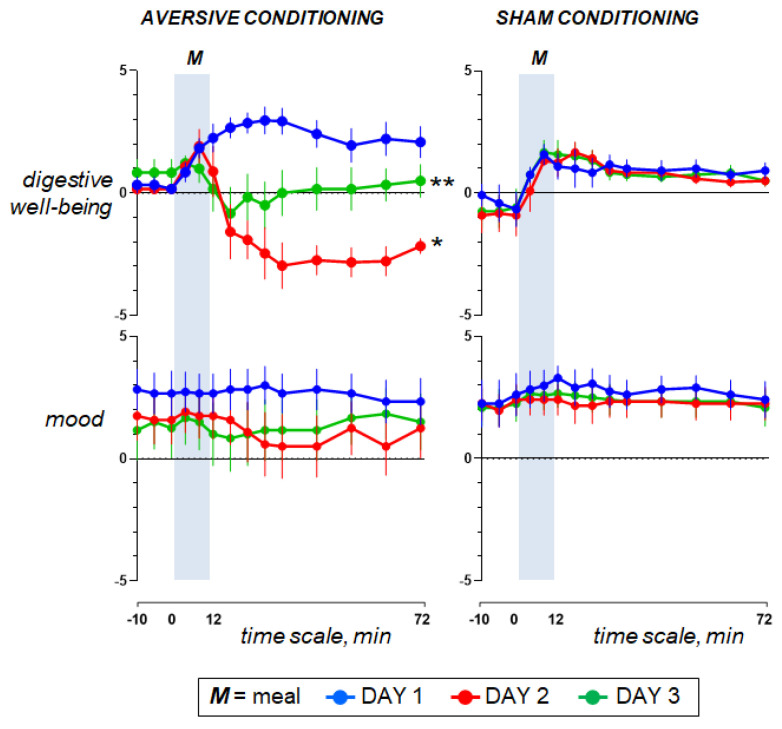
Hedonic sensations. Concomitant duodenal lipid infusion on day 2 impaired the postprandial response and the effect was significant for digestive well-being (effect measured as the change in the area under the curve on day 2 minus day 1; * *p* < 0.001 vs. sham infusion). Data on day 3 show that aversive conditioning in the test group (previous exposure to the aversive stimulus) significantly impaired postprandial well-being (effect measured as the change in the area under the curve on day 3 minus day 1; ** *p* = 0.004 vs. sham conditioning). Values represent mean ± SE.

Pre-ingestion. Before the probe meal (baseline fasting period), subjects reported hunger, neutral digestive well-being and positive mood without the sensations of abdominal fullness/bloating, discomfort/pain or nausea (Figure 2 and Figure 3).

**Figure 4:**
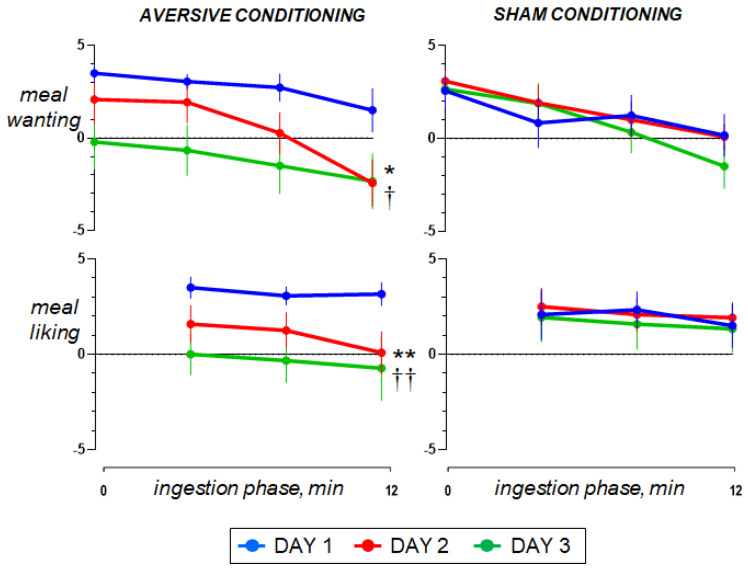
Reward value during meal ingestion. The comfort meal was served in 3 portions; meal wanting was measured before each serving and at the end of ingestion; meal liking was measured after each serving. On day 2, concomitant duodenal lipid infusion impaired the ingestive response (effect measured as the change in the area under the curve on day 2 minus day 1 vs. sham infusion; * *p* = 0.041 for meal wanting; ** *p* = 0.013 for meal liking). Data on Day 3 show that aversive conditioning in the test group (previous exposure to the aversive stimulus) significantly reduced the valence of the comfort meal (effect measured as the change in the area under the curve on day 3 minus day 1 vs. sham conditioning; ^†^ *p* = 0.023 for meal wanting; ^††^ *p* = 0.030 for meal liking). Values represent mean ± SE.

Ingestion phase. All participants ingested the meal at a fixed rate (12 min). Participants found the meal attractive at the initial presentation and liked it (positive meal wanting and meal liking; Figure 4). During meal ingestion satiety progressively increased, associated with mild fullness sensation and positive sensations of digestive well-being and mood, without abdominal discomfort or nausea (Figure 2 and Figure 3).

Postprandial phase. During the postprandial phase, these sensations gradually decayed (Figure 2 and Figure 3).

No significant differences in the sensations measured before, during and after ingestion were detected between groups.

### 3.3. Effect of Aversive Stimulation (Study Day 2 vs. Day 1)

Pre-ingestion and ingestion phase. The sensations measured before and during meal ingestion on the second study day were not different from those on the first day in both groups (Figure 2 and Figure 3), except for meal wanting and meal liking (Figure 4), which were reduced by duodenal lipid infusion in the test group, but were unaffected by sham infusion in the control group; the effect of lipid infusion in the aversive conditioning group (measured as the change in the area under the curve for Day 2 minus Day 1) was significantly different from that of sham infusion in the control group both for meal wanting (change by −36 ± 16 vs. 5 ± 12 score × min in controls; *p* = 0.041) and meal liking (change by −27 ± 9 vs. 2 ± 6 score × min in controls; *p* = 0.013).

Postprandial phase. In the control group, sham infusion on the second study day did not modify the postprandial experience as compared to the first study day. By contrast, concomitant duodenal lipid infusion in the test group induced a marked change in postprandial sensations, with an increase in satiety and bloating, a decrease in digestive well-being and mood and some degree of abdominal discomfort and nausea (Figure 2 and Figure 3). The effect of lipid infusion (measured as the change in the area under the curve on day 2 minus day 1) was significantly different from that of sham infusion for digestive well-being (change by −294 ± 34 vs. 25 ± 20 score × min in controls; *p* < 0.001), abdominal discomfort (change by 172 ± 65 vs. 0 ± 0 score × min in controls; *p* = 0.002) and nausea (change by 134 ± 28 vs. 0 ± 0 score × min; *p* = 0.002).

### 3.4. Effect of Conditioning (Study Day 3 vs. Day 1)

Pre-ingestion and ingestion phase. No significant differences were detected in the sensations measured before and during meal ingestion on the study day 3 as compared to day 1 in both groups (Figure 2 and Figure 3), except for meal wanting and meal liking (Figure 4). Meal wanting and liking were unaffected in the control group, but in the test group, previous exposure to the aversive stimulus (aversive conditioning) significantly reduced the valence of the comfort meal; the effect of conditioning (duodenal lipid or sham infusion on the previous study day), measured as the change in the area under the curve on day 3 minus day 1, was significantly decreased in the aversive conditioning group compared to the control group both for meal wanting (change by −62 ± 16 vs. −6 ± 9 score × min in controls; *p* = 0.023) and for meal liking (change by −43 ± 13 vs. −4 ± 5 score × min in controls; *p* = 0.030). Meal wanting and liking on day 3 were somewhat, but not significantly, lower than on day 2.

Postprandial phase. In the control group, sham conditioning (duodenal sham infusion on the previous day) did not modify the postprandial experience as compared to the first study day. By contrast, in the test group, aversive conditioning (duodenal lipid infusion on the previous day) significantly impaired postprandial well-being, and this was associated with a trend to increase in bloating and mild abdominal discomfort, without changes in satiety, nausea and mood (Figure 2 and Figure 3). The effect of lipid infusion on the previous day (measured as the change in the area under the curve on day 3 minus day 1) was significantly different from that of sham infusion for digestive well-being (change by −186 ± 68 vs. 16 ± 19 score × min in controls; *p* = 0.004), but not for the rest of the sensations: bloating changed by 74 ± 67 vs. −16 ± 40 score × min in controls (*p* = 0.235), abdominal discomfort by 43 ± 23 vs. 0 ± 0 score × min in controls (*p* = 0.074), satiety by −2 ± 50 vs. 82 ± 43 score × min in controls (*p* = 0.179), nausea by 6 ± 4 vs. 0 ± 0 score × min in controls (*p* = 0.181) and mood by 8 ± 27 vs. −12 ± 31 score × min in controls (*p* = 0.813).

### 3.5. Physiological Parameters 

#### 3.5.1. Responses to the Probe Meal before Conditioning (Study Day 1)

**Figure 5:**
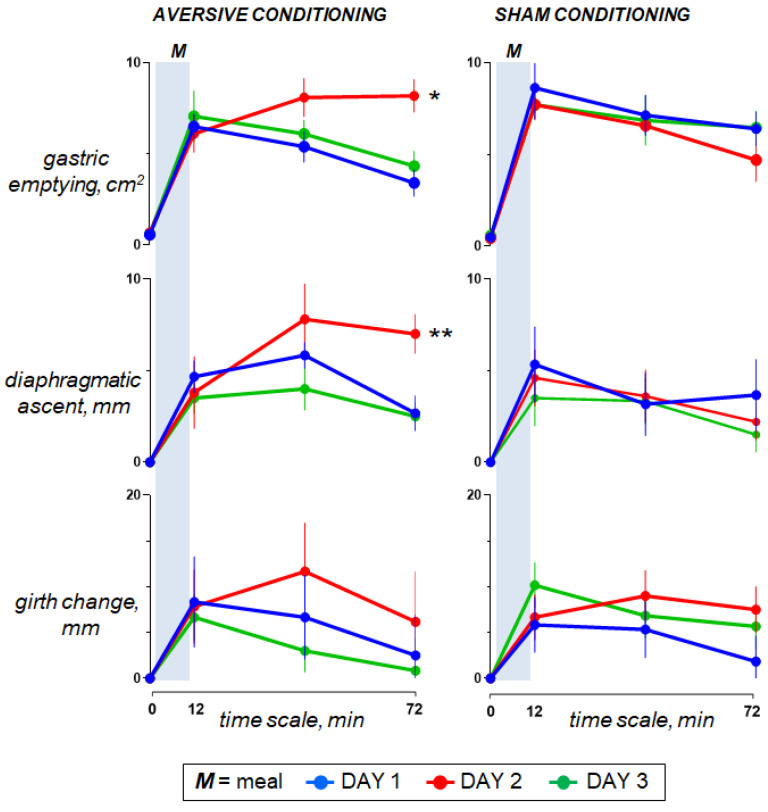
Digestive response to meal ingestion. On day 2, duodenal lipid infusion in the test group was associated with a sustained increase in antral cross-sectional area (delayed gastric emptying; * *p* < 0.001) and diaphragmatic ascent (prolonged abdominal accommodation; ** *p* = 0.002); effects measured as the changes in the area under the curve on day 2 minus day 1 vs. sham infusion. However, data for day 3 show that aversive conditioning in the test group (previous exposure to the aversive stimulus) did not induce significant effects. Values represent mean ± SE.

Ingestion of the probe meal was associated with gastric filling (increase in antral cross-sectional area) and abdominal accommodation (elevation of the diaphragm with limited increase in girth) (Figure 5).

#### 3.5.2. Effect of Aversive Stimulation (Study Day 2 vs. Day 1)

In the control group, sham infusion on the second study day had no effects on any of the physiological parameters as compared to the first study day. By contrast, in the test group, lipid infusion (effect measured as the change in the area under the curve in day 2 minus day 1 vs. sham infusion) was associated with gastric retention (more sustained increase in the antral cross-sectional area; *p* < 0.001) and abdominal accommodation (elevation of the diaphragm; *p* = 0.002), (Figure 5).

#### 3.5.3. Effect of Conditioning (Study Day 3 vs. Day 1)

Neither aversive nor sham conditioning had consistent effects on the physiological response to meal ingestion (Figure 5).

## 4. Discussion

Our study shows that pairing a pleasant meal with an experimentally-induced aversive sensation conditions the postprandial response to subsequent consumption of the same meal. Interestingly, aversive conditioning impaired the hedonic experience without significant impacts on homeostatic sensations or the physiological digestive response.

The target for conditioning was a comfort probe meal [17] that induced a pleasant and rewarding postprandial experience. The comfort probe meal was blindly paired with duodenal lipid infusion to induce a negative sensation of digestive well-being [27]. As expected [27,28], duodenal lipids induced a mild sensation of abdominal bloating, discomfort and nausea, as well as a reflex inhibition of gastric emptying with a prolonged residency of the meal in the stomach; gastric retention was associated with a sustained abdominal accommodation (elevation of the diaphragm) and sensation of satiety throughout the postprandial observation period.

Aversive conditioning (i.e., previous pairing of the comfort meal with lipid-induced aversive sensation) conditioned the subsequent postprandial response to the same meal, particularly affecting the reward experience. Various mechanisms may be involved in the impairment of the postprandial experience by conditioning.

In the first place, a satisfactory postprandial experience depends on a normal response of the digestive system, and conversely, digestive dysfunction deteriorates the postprandial experience [14], but conditioning did not affect the digestive function.

The conditioning paradigm used in the present experiments was analogous to that previously applied for conditioned taste aversion, pairing the rewarding meal with an aversive stimulus [29,30]. Remarkably, similar to conditioned taste aversion, postprandial conditioning was acquired after a single exposure [30], in contrast to the complex learning process with repeat pairing experiences required for other types of Pavlovian conditioning [6]. In previous studies, we showed that postprandial satisfaction is related to meal palatability [16], but a strong aversive taste was required to reduce postprandial well-being, to a much lesser extent than in the present study after conditioning.

Cognitive-emotive factors and expectations might be involved in conditioning the post-prandial experience. Indeed, a cognitive intervention (education) influenced the hedonic postprandial experience, without significant effects on homeostatic sensations [19], an effect similar to that produced by conditioning in the present study. Expectations are also important: mislabelled foods produce the effect expected by the (mis)information provided; for instance, a low-fat yoghurt mislabelled as high-fat induced similar symptoms to the real high-fat yoghurt in dyspeptic patients [31]. Anticipatory knowledge and attention have been shown to heighten visceral sensitivity and increase perception of intestinal stimuli; intestinal distention produced more intense perception when the stimuli were anticipated by a visual signal than when participants were distracted by a cognitive task [32].

## 5. Limitations

Our conditioning paradigm introduced a colour clue (coloured meal during and after conditioning versus non-coloured meal pre-conditioning), but we do not know whether conditioned postprandial dissatisfaction was selective to the colour or if it would also affect the non-coloured meal; indeed, other forms of conditioning express generalisation and affect related stimuli [33,34].

For this pilot study, a small sample size was included due to the complexity and invasiveness of the study; an interim sample size calculation justified no further inclusion for practical and ethical considerations, but once the concept is proven, a larger study with a less invasive methodology is indicated. Furthermore, only women were included and the effect of conditioning on men remains to be explored.

This pilot study, proving a new concept, opens a series of questions, particularly in relation to the specificity versus generalisation of the conditioned response, extinction interval and the relation between aversive stimulus/conditioned response [30,33,34], that remain to be addressed.

## 6. Conclusions and Inferences

Postprandial conditioning might have important implications and open relevant research avenues. Several conditions of great health impact, such as obesity, metabolic syndrome, diabetes or hypercholesterolemia, relate to consumption (or overconsumption) of specific foods, and in this context, aversive conditioning could be a tool to promote an avoidance behaviour.

The proof of aversive conditioning sustains the hypothesis of reward conditioning. If feasible, reinforcement of the postprandial reward and food valence could be applied to counteract natural neophobia (i.e., rejection of new or unknown foods) in children [30], and to promote ingestion in patients with anorexia and nutritional deficits, a common problem in oncological patients.

Patients with functional gut disorders, particularly with functional dyspepsia, complain of postprandial symptoms in the absence of a detectable cause and constitute about half of gastrointestinal consultations. Aversive food conditioning might be a mechanism of meal intolerance in these patients. Based on the present data, it could be speculated that an analogous technique could be applied to deconstruct aversive conditioning in these patients [33]; de-conditioning of food intolerances may have important applications as a mechanistic treatment in patients with food-related symptoms.

## Data Availability

The data presented in this study will be shared upon reasonable request from the corresponding author.
